# Omega-3/Omega-6 Long-Chain Fatty Acid Imbalance in Phase I Retinopathy of Prematurity

**DOI:** 10.3390/nu14071333

**Published:** 2022-03-23

**Authors:** Zhongjie Fu, Wenjun Yan, Chuck T. Chen, Anders K. Nilsson, Edward Bull, William Allen, Jay Yang, Minji Ko, John Paul SanGiovanni, James D. Akula, Saswata Talukdar, Ann Hellström, Lois E. H. Smith

**Affiliations:** 1 Department of Ophthalmology, Boston Children’s Hospital, Harvard Medical School, Boston, MA 02115, USA; zhongjie.fu@childrens.harvard.edu (Z.F.); edward.bull@childrens.harvard.edu (E.B.); william.allen@childrens.harvard.edu (W.A.); jay.yang@childrens.harvard.edu (J.Y.); minji.ko@childrens.harvard.edu (M.K.); james.akula@childrens.harvard.edu (J.D.A.); 2 Center for Brain Science, Department of Molecular and Cellular Biology, Harvard University, Cambridge, MA 02138, USA; wey334@g.harvard.edu; 3 Laboratory of Neurogenetics, National Institute on Alcohol Abuse and Alcoholism, Bethesda, MD 20814, USA; tzuhuan.chen@utoronto.ca; 4 Section for Ophthalmology, Department of Clinical Neuroscience, Institute of Neuroscience and Physiology, Sahlgrenska Academy, University of Gothenburg, 412 96 Gothenburg, Sweden; anders.k.nilsson@gu.se (A.K.N.); ann.hellstrom@medfak.gu.se (A.H.); 5 BIO5 Institute, Department of Nutritional Sciences, The University of Arizona, Tucson, AZ 85721, USA; jpsangio@arizona.edu; 6 Cardiometabolic Diseases, Merck Research Laboratories, Boston, MA 02115, USA; saswata.talukdar@merck.com

**Keywords:** retinal vessel, retinal neuron, retinopathy of prematurity, LCPUFA, adiponectin, hyperglycemia

## Abstract

There is a gap in understanding the effect of the essential ω-3 and ω-6 long-chain polyunsaturated fatty acids (LCPUFA) on Phase I retinopathy of prematurity (ROP), which precipitates proliferative ROP. Postnatal hyperglycemia contributes to Phase I ROP by delaying retinal vascularization. In mouse neonates with hyperglycemia-associated Phase I retinopathy, dietary ω-3 (vs. ω-6 LCPUFA) supplementation promoted retinal vessel development. However, ω-6 (vs. ω-3 LCPUFA) was also developmentally essential, promoting neuronal growth and metabolism as suggested by a strong metabolic shift in almost all types of retinal neuronal and glial cells identified with single-cell transcriptomics. Loss of adiponectin (APN) in mice (mimicking the low APN levels in Phase I ROP) decreased LCPUFA levels (including ω-3 and ω-6) in retinas under normoglycemic and hyperglycemic conditions. ω-3 (vs. ω-6) LCPUFA activated the APN pathway by increasing the circulating APN levels and inducing expression of the retinal APN receptor. Our findings suggested that both ω-3 and ω-6 LCPUFA are crucial in protecting against retinal neurovascular dysfunction in a Phase I ROP model; adequate ω-6 LCPUFA levels must be maintained in addition to ω-3 supplementation to prevent retinopathy. Activation of the APN pathway may further enhance the ω-3 and ω-6 LCPUFA’s protection against ROP.

## 1. Introduction

Retinopathy of prematurity (ROP) is a common complication of premature birth and a leading cause of blindness in children worldwide [1]. ROP, a two-phased disease, occurs in premature infants born with an incompletely vascularized retina. In Phase I ROP, vessels do not grow and the retina becomes hypoxic causing uncontrolled vascular growth (Phase II ROP). Current treatments controlling oxygen-regulated factors such as vascular endothelial cell growth factor (VEGF) focus on the late vision-threatening uncontrolled retinal vessel growth (Phase II ROP) [2,3,4]. However, the underlying problem of delayed retinal vascular development, which initiates the disease, remains unaddressed. A major (understudied) risk factor for ROP is perinatal hyperglycemia [5,6,7,8,9,10,11,12].

In the last two decades, clinical investigations have shown that postnatal hyperglycemia in the first few weeks of life, independent of oxygen, is strongly associated with ROP. Hyperglycemia commonly occurs in ~80% of preterm infants with a birth weight of less than 750 g and ~45% with a birth weight of less than 1000 g [13]. Infants with hyperglycemia are also smaller and more immature than those with normoglycemia [13]. Low gestational age is associated with hyperglycemia in extremely low birth weight infants in the first 2 weeks of life, and hyperglycemia increases the incidence of ROP [14]. Each 10 mg/dL increase of mean blood glucose correlates with an increased ROP risk [7]. Infants with ROP also experience more days of hyperglycemia. A higher number of mean days of hyperglycemia significantly associates with ROP [8]. Hyperglycemia in the first week of life is independently associated with ROP after adjustment for gestational age, oxygen, respiratory support, and poor weight gain [10]. The frequency of hyperglycemia in infants with ROP is six times higher than those without ROP [6]. Hyperglycemia during the first 10 and 30 days of life also predicts ROP severity [12]. However, experimental investigations of hyperglycemia on ROP progression are lacking, because the commonly used oxygen-induced retinopathy model mimics the oxygen-regulated aspects of ROP but not the hyperglycemic aspect. In order to better understand the role of hyperglycemia in retinal vascular development, we recently established a mouse model of hyperglycemia-associated Phase I retinopathy with suppression of normal vascular development. Neonatal hyperglycemia is induced through daily administration of streptozotocin (STZ) from postnatal day 1 to 9 [15]. Induction of postnatal hyperglycemia delays retinal vascularization, modeling Phase I ROP. There are limitations to this model. As hyperglycemia starts around P8 when the superficial vascular net has almost completely formed. Thus we do not see delayed development in the superficial network formation but only in the deep retinal vessel growth, which follows the superficial vascular formation in mice (and also in human). We found that VEGF was not the primary driver of the vessel growth delay in hyperglycemia-associated Phase I retinopathy, suggesting additional evidence of other growth factors involved in ROP pathogenesis. Another limitation is that our model only reflects hyperglycemia resulting from insulin deficiency but not insulin resistance which is also found in premature infants [16].

In premature infants, there is a lack of both ω-3 and ω-6 LCPUFA (docosahexaenoic acid, DHA, and arachidonic acid, AA) normally transferred from the mother to the fetus in utero during the third trimester [17,18]. Several clinical studies evaluated the effects of supplementing DHA on the development of ROP and visual function in premature infants. However, the results were inconsistent. In some ROP studies, supplementing premature infants with a fish-oil containing lipid emulsion (rich in ω-3 LCPUFAs) vs. soybean oil (rich in ω-6 linoleic acid) or olive oil-based lipid emulsion (rich in oleic acid) reduced the risk for severe ROP [19,20,21]. Preterm infants receiving a mixed oil lipid emulsion with fish oil vs. an olive oil emulsion had the same incidence of any ROP or severe ROP [22]. Hellström et al. found that adequate AA levels are needed for DHA protection against severe ROP [23].

With respect to visual function, supplementing premature infants with formula at 0.32% DHA and 0.64% AA of total fatty acids improved visual acuity at 12 months of age [24]. However, increasing DHA to 0.64% or 0.96% (with 0.64% AA supplementation) was not associated with additional improvement of visual acuity [24]. Premature infants receiving human milk from mothers ingesting tuna oil (rich in ω-3 DHA and eicosapentaenoic acid EPA) vs. soy oil (rich in ω-6 linoleic acid), or receiving formula supplemented with high DHA (1% DHA vs. ~0.3% DHA), had better visual acuity at 4 months corrected age [25]. In another study, very preterm infants supplemented with human milk with 1% DHA vs. 0.2–0.3% DHA in the first months of life did not have an improved visual function at 7 years of age [26]. The reasons behind these inconsistent observations have yet to be uncovered. A better understanding of the role ω-3/ω-6 LCPUFAs in retinal development will help determine the most effective lipid intervention for protection against ROP in premature infants.

Our previous studies demonstrated that low APN levels in premature infants correlated with delayed retinal vascularization in Phase I ROP [15]. APN mediates the protective effects of ω-3 LCPUFA against retinal vascular pathology in mice modeling proliferative retinopathy [27,28]. ω-3 LCPUFA also further increases APN levels [28,29]. The ω-3 LCPUFA DHA and ω-6 LCPUFA AA are essential lipids and critical for the growth and function of the brain, retina, and vascular system [30,31]. In this study, we evaluated the relationship between APN and retinal lipid composition as well as the ω-3/ω-6 LCPUFA balance as they relate to normal retinal vascularization in Phase I ROP.

In this study using the hyperglycemia-associated Phase I ROP model, we investigated the role of ω-3 LCPUFA in retinal development with diets enriched with either ω-3 or ω-6 LCPUFA lipids (ω-3 diet with 1% DHA plus 1% EPA and no AA, vs. ω-6 diet with 2% AA and no DHA or EPA) to expand our current knowledge of retinal LCPUFA imbalance in retinal neurovascular disorders. We also found a large change in retinal LCPUFA content in APN-deficient mice with Phase I ROP.

## 2. Materials and Methods

### 2.1. Study Approval

All animal studies adhered to the Association for Research in Vision and Ophthalmology Statement for the Use of Animals in Ophthalmic and Vision Research and were approved by the Institutional Animal Care and Use Committee at Boston Children’s Hospital (19-04-3913R).

### 2.2. Neonatal Mouse Model of Hyperglycemia-Associated Phase I ROP

In the mouse hyperglycemia-associated retinopathy model (referred as Phase I ROP through the text) with delayed retinal vascularization, hyperglycemia was induced with STZ (50 mg/kg) administered intraperitoneally daily from postnatal day P1 to P9 as previously described [15]. STZ-treated mice vs. non-STZ-treated mice had lower body weight which can affect retinal vascular development. Therefore, the litter size in the STZ-treated group was limited to six pups vs. non-STZ-treated (eight to nine pups per litter) mice to achieve equal weight gain [15]. Intravitreal injection of STZ has no direct impact on retinal vessel growth [15]. In mouse retinas, the superficial layer forms from P1 to P10, the deep layer from P8 to P12, and the intermediate layer from P14 to P20, each of which grows from the central to the peripheral retina [32]. At P10, the mice were euthanized with ketamine/xylazine and retinas were collected. Blood samples were collected for serum. Body weight and blood glucose levels were recorded. Both female and male pups were used.

For dietary LCPUFA supplementation, C57BL/6J nursing dams and pups (wild-type (WT)) and APN-deficient (*Apn^−/−^*) mice (Jackson Laboratory, #008195, backcrossed to C57BL/6J for 11 generations) were fed with completely defined isocaloric rodent feeds with 10% (*w*/*w)* high oleic safflower oil containing either 2% ω-3 LCPUFAs (1% DHA and 1% EPA) or 2% ω-6 LCPUFAs (AA, control for ω-3) from birth or P6 [27,28,33,34,35]. The DHA, EPA, and AA oils were from DSM (Heerlen, The Netherlands) and the customized feed was produced at Research Diets, Inc. (New Brunswick, NJ, USA) The diet number and the composition of the feed is shown in Table S1. The dietary fatty acid compositions were quantified to confirm the ω-3 and ω-6 diet quality (data not shown) using the same methods for retinal lipid composition analysis described below. ω-3- and ω-6-enriched diets with all other nutrients defined were compared. The retinal ω-3 and ω-6 lipid composition in the pups reflects the mother’s dietary intake of lipids, as we have previously reported [33].

### 2.3. Quantification of Retinal Vasculature

The retinas were stained with fluorescent Griffonia/Bandeiraea simplicifolia Isolectin B4 (10 μg/mL in 1 mM CaCl_2_ in phosphate buffered saline (PBS), Molecular Probes, I21413) overnight at room temperature. To quantify the retinal vascular network, 4–5 images between the optic nerve head and the leading edge of vessels in the deep and superficial vascular layers were taken at 200× magnification on a Zeiss AxioObserver Z1 microscope as previously described [15]. The images were then analyzed in Image J. The background staining was cleaned manually first and the image was converted to 8-bit. The retinal vascular network was analyzed using the Angiogenesis analyzer plugin. The number of meshes and total vessel length per field were compared between groups. 

### 2.4. Single-Cell Transcriptomics 

Retinas from Phase I ROP (*n* = 3 mice) vs. vehicle PBS- (*n* = 3 mice) treated, or ω-3 (*n* = 2 mice) vs. ω-6 (*n* = 3 mice) diet-fed C57BL/6J mouse littermates from the hyperglycemia-associated Phase I ROP model were carefully dissected with their anterior segment and retinal pigment epithelium cells removed. Retinal single-cell suspensions were prepared using the Worthington papain dissociation system following the manufacturer’s protocol [36,37]. A retinal cell barcoded library was prepared at the Single Cell Core at Harvard Medical School (HMS) [38,39], sequenced at the Biopolymer Facility at HMS, and aligned to the mouse genome at the Harvard Chan Bioinformatics Core. 

Sequencing of the libraries was conducted at the HMS Biopolymer Facility using an Agilent 4200 Tapestation instrument as previously described [40]. A corresponding Agilent High Sensitivity D1000 ScreenTape assay was used to visualize the libraries and check that the size and concentrations of the libraries matched the expected product. The functional concentration was confirmed with qPCR with the KAPA Library Quantification kit, which uses primers complementary to the sequencing flowcell oligos. All samples were normalized in equimolar ratio for one final pool, using molarity values from the Agilent High Sensitivity D1000 ScreenTape assay. The pool was denatured and loaded (at 2.5 pM) onto an Illumina NextSeq 500 instrument, with a High-Output 75-cycle kit to obtain paired-end reads (read 1 = 61 cycles; Read 2 = 14 cycles). The basecall files were then demultiplexed using the Harvard BPF Genomics Core’s pipeline as previously reported [41]. Reads were processed with the inDrop v3 pipeline implemented in bcbio-nextgen version 1.2.4-76d5c4b. Dual cellular barcodes, sample barcodes, and UMIs were identified using umis [42]. Cells with fewer than 500 total reads assigned to them were excluded from further analysis. Reads were assigned to Mus musculus transcripts (GRCm38 (mm10) release M23) using Rapmap [43] and counts of reads per transcript per unique UMI were generated for each cell. The generated gene count matrix was used for subsequent analysis. Raw data were deposited at Gene Expression Omnibus (GEO) and can be accessed with accession number GSE198785.

The downstream clustering analysis was performed using R package ‘Seurat’ (version 3.2.2) [44], genes and cells were filtered so only cells with more than 300 genes detected or genes expressed in more than 10 cells were kept. The filtered gene count matrix was log normalized with a scale factor of 10,000 to generate the gene expression matrix. The top 2000 high variable genes (HVGs) were selected using the ‘vst’ method from the Seurat function FindVariableFeatures. The gene expression matrix was scaled and principal component analysis was performed on all the HVGs of the scaled data and the top 100 principal components were computed. The first 75 principal components were used for clustering and UMAP visualization. Computed cell clusters at a resolution of 0.2 were assigned into major cell classes in the retina based on canonical markers [45]. Differentially expressed genes were identified using ‘MAST’ methods (MASTClassic) [46]. 

The gene enrichment analysis was performed using the ‘enrichGO’ function from R package ‘clusterProfiler’ and the mouse annotation database ‘EnsDb.Mmusculus.v79’. The *p*-value was adjusted using the Benjamini—Hochberg method with a cutoff of 0.05 on the enrichment tests. 

### 2.5. Real-Time PCR

RNA was prepared from mouse retinas as we previously reported [15,27]. Retinas were lysed with QIAzol lysis reagent and 20% chloroform was added to promote the separation of RNA from DNA and protein. RNA was extracted using a PureLink^®^ RNA Mini Kit (#12183018A, Ambion). RNA was then reverse transcribed using iScript^TM^ cDNA synthesis kit (#1708891, Bio-Rad, Hercules, CA, USA). qPCR was performed for insulin-like growth factor 1 (*Igf1*)*:* 5′-GGCTCCAGCATTCGGAGGGC-3′, 5′-CGCTGGGCACGGATAGAGCG-3′; *Igf1r:* 5′-GTGGGGGCTCGTGTTTCTC-3′, 5′-GATCACCGTGCAGTTTCCCA-3′; *Kdr:* 5′-CAAACCTCAATGTGTCTCTTTGC-3′, 5′-AGAGTAAAGCCTATCTCGCTGT-3′; *Vegfa:* 5′-GGAGACTCTTCGAGGAGCACTT-3′, 5′-GCGATTTAGCAGCAGATATAAGAA-3′. *AdipoR1:* 5′-TCTTCGGGATGTTCTTCCTGG-3′, 5′-TTTGGAAAAAGTCCGAGAGACC-3′. Quantitative real-time PCR was generated with the SYBR Green Master mix kit using an Applied Biosystems 7300 Sequence Detection System. Gene expression was normalized to the housekeeping gene *CyclophilinA:* 5′-CAGACGCCACTGTCGCTTT-3′; 5′-TGTCTTTGGAACTTTGTCTGCA-3′ using the ΔΔCt method. The relative mRNA levels were calculated as the ratio of change versus control group.

### 2.6. Electroretinography (ERG)

Hyperglycemia was induced in C57BL/6J mice with intraperitoneal injection of 25 mg/kg STZ daily from P2 to P12. The nursing dams were fed a ω-3 or ω-6 LCPUFA diet after giving birth. Retinal neuronal function was examined with ERG using a Colordome Ganzfeld stimulator and Epsion E2 amplifier (Diagnosys LLC, Lowell, MA, USA) as we previously described [15,36,47]. At P30, mice were dark-adapted overnight. On the second day, the mice were anesthetized with ketamine/xylazine, and their pupils were dilated with Cyclomydril (Alcon, Fort Worth, TX, USA). The stimuli were “green” light-emitting diode flashes of doubling intensity from ~0.0064 to ~2.05 cd·s·m^−2^ and then “white” xenon-arc flashes from ~8.2 to ~1050 cd·s·m^−2^. The rod photoreceptor function was quantified by fitting the free parameters in a model of the biochemical processes involved in the activation of phototransduction to the electroretinographic *a*-waves [48,49,50]. The postreceptor activity (e.g., bipolar cell function) was examined by fitting the Naka-Rushton equation [51] to the response-vs-intensity relationship of the *b*-wave. Total retinal sensitivity (*Sm*) was estimated as previously described [52]. The ERG data are presented as the log change from the ω-6 only diet-fed mice (ΔLogControl). 

### 2.7. Western Blot

A total of 1 µL mouse serum was incubated with Laemmli’s SDS sample buffer (BP-110R; Boston BioProducts Inc., Milford, MA, USA) for one hour at room temperature to maintain the secondary structure of APN as shown before [28]. The lysates were then loaded onto an SDS-PAGE gel and transferred onto a nitrocellulose membrane. The membrane was blocked with 5% BSA and incubated overnight with primary antibody APN (1:1000, AF1119; R&D, Minneapolis, MN, USA). The protein bands were detected by using corresponding horseradish peroxidase–conjugated secondary antibodies (1:5000) and enhanced chemiluminescence (ECL, Pierce, Waltham, MA, USA). The digital images were visualized with a Bio-Rad ChemiDoc Touch Imaging System and the band intensity was quantified with Image J. 

### 2.8. Lipidomic Fatty Acid Profiling of Mouse Retinas

P10 *Apn^−/−^* and C57BL/6J retinas under normoglycemic and hyperglycemic conditions were collected for retinal lipid composition analysis. Six to eight retinas were combined as *n* = 1 for lipid analysis. Total retinal lipids were extracted with chloroform, methanol, and 0.88% KCl (2:1:0.75 by volume). Individual phospholipid pools were isolated by thin-layer chromatography (TLC) using TLC H-plates (Analtech; Newark, DE, USA). Choline glycerophospholipids (ChoGpl) and ethanolamine glycerophospholipids (EtnGpl) were fractionated along with authentic standards (Avanti; Alabaster, AL, USA) using chloroform, methanol, 2-propanol, 0.25% KCl (*w*/*v*), and triethylamine (30:9:25:6:18 by volume). Fractionated bands were sprayed with 8-anilino-1-naphthalene sulfonic acid (0.1% *w*/*v*), visualized under UV light, and collected in a test tube with a known quantity of heptadecanoic acid (17:0) standard. Fatty acids from each phospholipid pool were converted into fatty acid methyl esters (FAME) with 14% boron trifluoride-methanol. ChoGpl and EtnGpl were converted at 100 °C for 1 h. FAME were quantified by gas chromatography-flame ionization detection (Agilent 7890A gas chromatograph coupled with fame ionization detector; Agilent Technologies Inc.; Santa Clara, CA, USA). The concentration of each fatty acid was quantified by peak comparison to 17:0 (heptadecanoic acid) standard. A heatmap was generated using the ratio of change in percent mol with Morpheus, Broad Institute. 

### 2.9. Statistical Methods

Researchers were blinded to the treatment conditions. Two-tailed unpaired *t*-test, or ANOVA test was used for comparison of results as specified in the figure legends (Prism v7.0; GraphPad Software, Inc., San Diego, CA, USA). *p* < 0.05 was considered as statistically significant.

## 3. Results

### 3.1. Dietary ω-3 LCPUFA Promoted Retinal Vessel Growth in Phase I ROP

**Figure 1:**
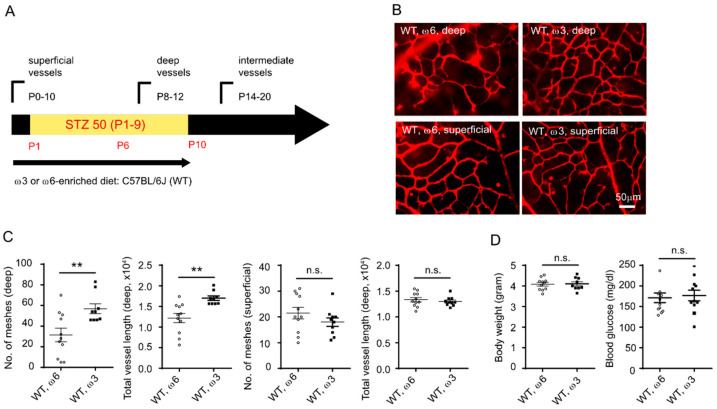
Dietary ω-3 vs. ω-6 LCPUFA supplementation from birth promoted retinal vessel growth in Phase I ROP mice. (**A**) Schematics of hyperglycemic induction in C57BL/6J mice. An amount of 50 mg/kg STZ was i.p. injected daily from P1 to P9. The mice were fed a ω-3 or ω-6 LCPUFA-enriched diet from birth until P10 when the retinas were collected for analysis. (**B**) Representative images of retinal vessels in P10 C57BL/6J STZ-mice fed on ω-3 or ω-6 LCPUFA-enriched diet from birth. Retinal vessels were stained with isolectin. Scale bar, 50 μm. (**C**) ω-3 versus ω-6 LCPUFA-enriched diet promoted retinal vascular network formation at P10 STZ. *n* = 9–11 eyes. Unpaired *t*-test. ** *p* < 0.01, n.s., no significance. Data was represented as mean ± SEM. (**D**) Comparable body weight and blood glucose in ω-3 versus ω-6 LCPUFA-fed C57BL/6J mice. *n* = 9–11 mice. Unpaired *t*-test. n.s., no significance. Data are represented as mean ± SEM.

**Figure 2:**
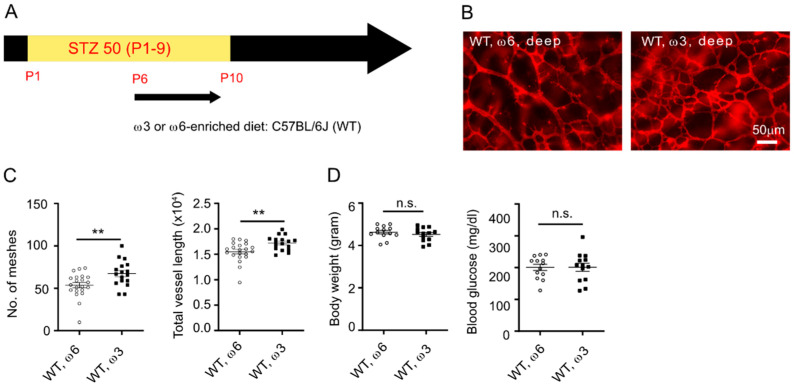
Dietary LCPUFA directly promoted deep retinal vessel growth in Phase I ROP. (**A**) Schematics of hyperglycemic induction in C57BL/6J mice. An amount of 50 mg/kg STZ was i.p. injected daily from P1 to P9. The mice were fed a ω-3 or ω-6 LCPUFA-enriched diet from P6 until P10 when the retinas were collected for analysis. (**B**) Representative images of deep retinal vessels in P10 C57BL/6J STZ mice fed a ω-3 or ω-6 LCPUFA-enriched diet. Retinal vessels were stained with isolectin. Scale bar, 50 μm. (**C**) ω-3 versus ω-6 LCPUFA-enriched diet promoted retinal vascular network formation at P10. *n* = 17–21 eyes. Unpaired *t*-test. ** *p* < 0.01. Data are represented as mean ± SEM. (**D**) Comparable body weight and blood glucose levels in ω-3 versus ω-6 LCPUFA-fed C57BL/6J mice. *n* = 13 mice. Unpaired *t*-test. n.s., no significance. Data are represented as mean ± SEM.

We have previously reported that hyperglycemia causes delayed development of the deep retinal vasculature in the Phase I ROP mouse model [15]. To examine the impact of ω-3 LCPUFA on retinal development, we fed the STZ-induced C57BL/6J (WT) with defined diets enriched with either 2% ω-3 LCPUFA (1% DHA and 1% EPA, no AA) or control 2% ω-6 LCPUFA (2% AA, no DHA or EPA, isocaloric control diet) from birth to P10 (Figure 1A). In normoglycemic mice, dietary supplementation with ω-3 versus ω-6 LCPUFA produced denser deep and superficial vascular networks (Figure S1). In hyperglycemic mice (with onset of hyperglycemia after formation of the superficial vascular network), dietary supplementation with ω-3 versus ω-6 LCPUFA resulted in denser deep vascular patterning and no significant difference in the superficial vasculature (Figure 1B,C). We found that supplementation with ω-3 versus ω-6 LCPUFA diet right before deep retinal vessel growth starts [32] (P6 to P10) had a direct impact on promoting deep retinal vascular patterning (Figure 2A–D). We also observed that the deep vascular network in Phase I ROP mice fed a ω-6 LCPUFA diet from P6 to 10 was denser than those fed from birth to P10. We speculated that the earlier exposure of the nursing dam to the unbalanced LCPUFA diet without ω-3 LCPUFA led to poorer retinal vasculature development in the Phase I ROP model. Comparable body weight and blood glucose was observed in ω-3 or ω-6 LCPUFA fed C57BL/6J mice, suggesting that the differences in retinal vascular development were not due to overall body growth and modulation of hyperglycemia (Figure 1 and Figure 2).

### 3.2. Dietary ω-6 LCPUFA Facilitated Retinal Neuronal Maturation

**Figure 3:**
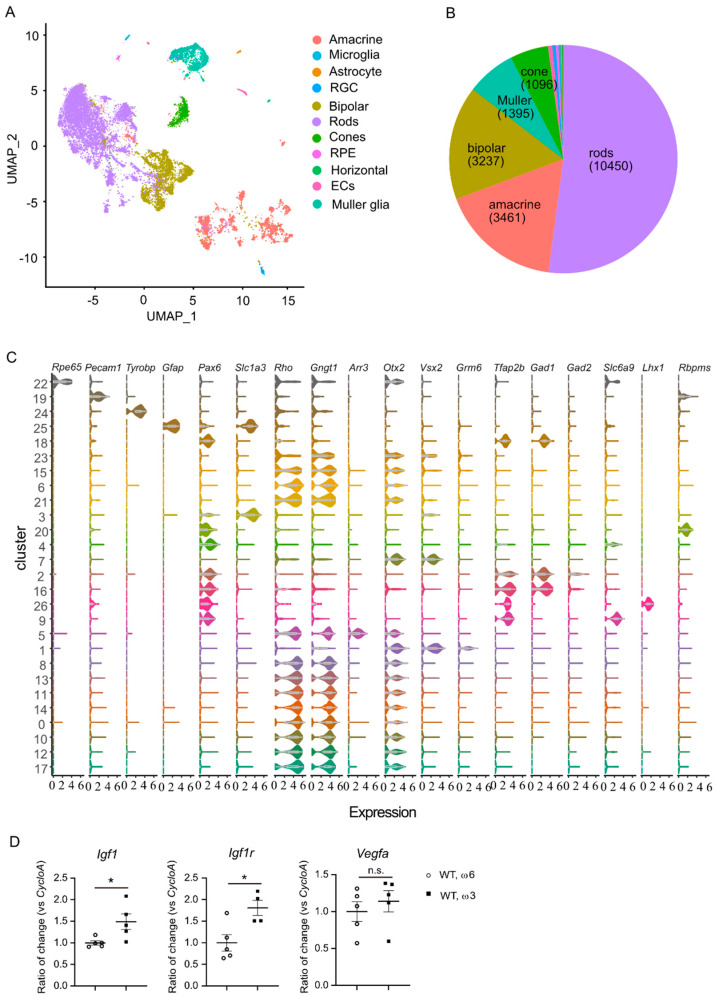
Single-cell analysis of retinas from ω-3 and ω-6-enriched diet-fed Phase I ROP mice. (**A**) UMAP visualization of the dataset, color represents cell classes. (**B**) Proportions of each cell class captured in the dataset, the exact cell number of the top five classes are labeled in the pie chart. (**C**) Visualization of canonical marker gene expressions in each cluster using violin plot. On the x-axis is the expression level of the genes marked on top of each column, while on the y-axis is the clusters computed using resolution of 0.2 (see Methods). (**D**) **A** ω-3 versus ω-6 LCPUFA-enriched diet increased gene expression of total retinal *Igf1* and *Igf1r,* not *Vegfa*, identified with qPCR. *n* = 4–5 mice. Unpaired *t*-test. * *p* < 0.05, n.s., no significance. Data are represented as mean ± SEM.

To further understand the impact of ω-3 versus ω-6 LCPUFA on retinal cellular function in Phase I ROP, we examined differentially expressed genes in the major retinal cells (rods, cones, Müller glia, bipolar cells, and amacrine cells) by comparing ω-3 vs. ω-6 LCPUFA diets in Phase I ROP mice using single-cell transcriptomics data. Cells from all groups at P10 were pooled, subjected to RNA sequencing, and then clustered and assigned into major cell classes based on the expression of canonical markers. Differentially expressed genes between the two conditions were therefore assessed in each cell class (See Methods for details) (Figure 3A–C). The expression of *Igf1* (logFC = 0.34834 in bipolar cells, *p* < 0.01) and *Igf1r* (logFC = 0.34605, *p* < 0.001 in rods, logFC = 0.29324, *p* < 0.05 in cones, logFC = 0.29486, *p* < 0.01 in bipolar cells, logFC = 0.28019, *p* < 0.05 in amacrine cells, logFC = 0.27061, *p* < 0.05 in RGCs) was higher in ω-3 versus ω-6 LCPUFA-fed mice, in line with increased total mRNA levels of *Igf1* and *Igf1r* in ω-3-fed mouse retinas validated with qPCR (Figure 3D). There were no significant differences in *Vegfa* expression identified both in single-cell analysis and qPCR, in line with our previous observations that dietary supplementation of ω-3 vs. ω-6 LCPUFA does not change retinal *Vegfa* expression [27,33]. These finding suggested that single-cell analysis was a reliable approach for quantifying gene expression levels in retinas of the ω-3 versus ω-6 LCPUFA-fed mice. 

We found a higher expression of genes involved in visual development, including visual perception, eye development, axon development, and synapse organization related gene ontology terms in rod, cone, bipolar, and amacrine cell clusters of Phase I ROP vs. normal control retinas (Figure S2A,B). In mouse retinas, neurons complete proliferation and differentiation around two weeks after birth [53]. The current data suggest a possible delay in neural retinal development in Phase I ROP vs. control retinas at P10. We also observed a significantly lower expression of genes involved in metabolic pathways in rod, cone, bipolar, and amacrine cell clusters in Phase I ROP vs. control retinas (Figure S3A,B). Taken together, the Phase I ROP vs. normal control retinas had a delay in neural proliferation and differentiation, accompanied by a lower metabolism. Our findings were consistent with our previously reported decreased retinal neuronal signals and reduced retinal thickness as well as decreased metabolic gene expression in photoreceptors in Phase I ROP vs. control retinas [15]. 

**Figure 4:**
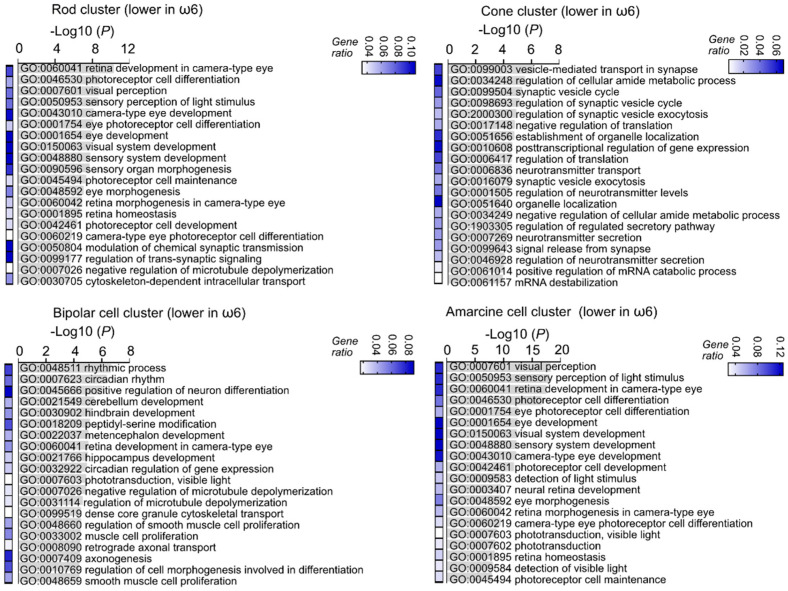
ω-6 vs. ω-3 accelerated neural retinal maturation identified with single-cell analysis. The lower expression of genes in the rod, cone, bipolar, and amacrine cell clusters of ω-6 versus ω-3 LCPUFA-fed Phase I ROP mice were associated with visual signal transduction (synapse-related, circadian rhythm) and eye development related gene ontology terms. Adjusted *p*-values for enriched gene ontology (GO) terms are shown in bar graphs (*p* < 0.05). Gene ratio for each pathway is shown in heatmap.

**Figure 5:**
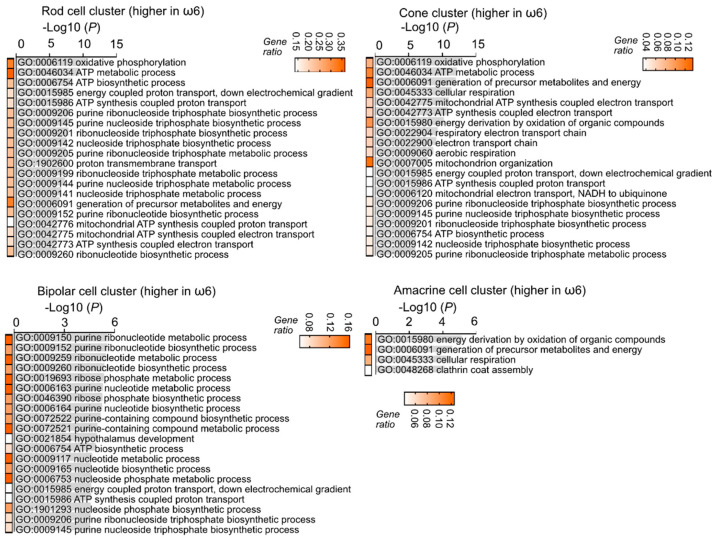
ω-6 vs. ω-3 increased retinal neuronal metabolism identified with single-cell analysis. The higher expression of genes (top 20) in the rod and cone cluster of ω-6 vs ω-3 diet-fed Phase I ROP mice were associated with energy production related gene ontology terms. The higher expression of genes in the bipolar cell cluster of ω-6 versus ω-3 LCPUFA-fed Phase I ROP mice were associated with purine ribonucleotide (components of DNA, RNA, adenosine triphosphate (ATP), guanosine triphosphate (GTP), cyclic adenosine monophosphate (cAMP), nicotinamide adenine dinucleotide (NADH), and coenzyme A) metabolic and biosynthetic process related gene ontology terms. The higher expression of genes in the amacrine cell cluster of ω-6 versus ω-3 LCPUFA-fed Phase I ROP mice were associated with energy production related gene ontology terms. Adjusted *p*-values for enriched gene ontology (GO) terms are shown in bar graphs (*p* < 0.05). Gene ratio for each pathway is shown in heatmap.

Interestingly, the ω-6 vs. ω-3 LCPUFA diet was associated with a lower expression of visual signal transduction and visual development genes in rod, cone, bipolar, and amacrine cell clusters (Figure 4), suggesting a faster completion of retinal neuronal proliferation and differentiation in ω-6 vs. ω-3-fed Phase I ROP mice. Gene ontology analysis showed that ω-6 vs. ω-3 LCPUFA diet had a higher gene expression of metabolic pathways in rods, cones, bipolar, and amacrine cell clusters (Figure 5), in line with a higher metabolic demand in more mature neural retinas. Our observations suggested that ω-6 LCPUFA is essential in neural retinal development. We therefore predicted that loss of ω-6 LCPUFA in the diet may cause retinal dysfunction in the long term. 

### 3.3. Müller Glia Exerted Compensatory Response to Delayed Retinal Maturation

In the Müller glial cluster, there was an increased expression of genes involved in gliogenesis, axon development, and angiogenesis in Phase I ROP vs. control retinas (Figure S2C). Müller glial differentiation in mouse retinas starts after birth and peaks at the first postnatal week [53]. The higher expression of genes involved in gliogenesis suggested a delay in maturation in Müller glia in Phase I ROP retinas, in line with a lower expression of genes involved in energy production-related pathways in Müller glia (Figure S3C). We also recently reported that Müller glia modulate their gene profile in response to photoreceptor stress. Müller glia respond to degenerating photoreceptors with an increased expression of genes involved in axon development, synapse formation, and angiogenesis [36] as compensatory effects. In addition, Müller glial cell-derived *Vegfa* is a driving force of retinal angiogenesis [54]. Taken together, our findings suggested that Müller glia experienced a developmental delay and exerted compensatory responses to facilitate retinal neural and vascular growth in Phase I ROP.

**Figure 6:**
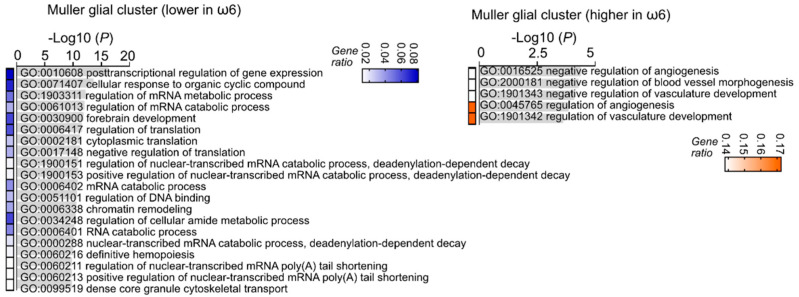
ω-6 versus ω-3 increased Müller glial angiogenic factor expression with single-cell analysis. In the Müller glial cell of ω-6 vs. ω-3 LCPUFA-fed Phase I ROP mice, the lower expression of genes was associated with post-transcriptional regulation, and the higher expression of genes was associated with regulation of angiogenesis and vasculature development related gene ontology terms. Adjusted *p*-values for enriched gene ontology (GO) terms are shown in bar graphs (*p* < 0.05). Gene ratio for each pathway is shown in heatmap.

The ω-6 vs. ω-3 LCPUFA diet showed suppression of angiogenesis in the Müller glial cluster in the Phase I ROP model (Figure 6). For example, ω-6 diet-fed mice had a higher expression of antiangiogenic secreted protein acidic and rich in cysteine (Sparc) [55] in the Müller glial cluster (logFC = 0.62687, *p* < 0.001). This observation corresponds to ω-6 vs. ω-3 LCPUFA-inhibition of retinal vessel growth (Figure 1 and Figure 2). 

### 3.4. Dietary ω-6 LCPUFA Improved Retinal Neuronal Function

**Figure 7:**
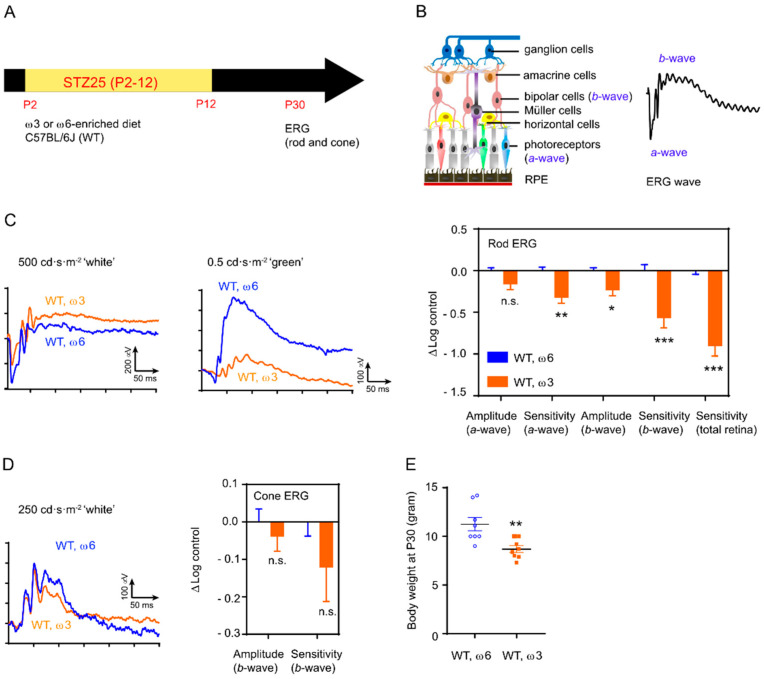
Shortage of dietary ω-6 LCPUFA delayed retinal functional development in Phase I ROP at P30. All ERG data are presented as the log change from control (ΔLog control) and normalized to that from ω-6-diet-fed group. Data are presented as mean ± SEM. (**A**) Schematics of hyperglycemic induction in C57BL/6J mice. An amount of 25 mg/kg STZ was i.p. injected daily from P2 to P12. The mice were fed on ω-3 or ω-6 LCPUFA-enriched diet from P1 until P30. (**B**) Schematics of ERG wave and corresponding retinal neurons: a-wave (photoreceptors), b-wave (bipolar cells). (**C**) ω-3 versus ω-6 LCPUFA diet decreased rod pathway responses in Phase I ROP mice. Representative rod ERG plots with ‘white’ (for maximal a-wave) and ‘green’ (for maximal b-wave) light stimulation are shown. *n* = 13–14 eyes. Multiple *t*-test. * *p* < 0.05, ** *p* < 0.01, *** *p* < 0.001, n.s., no significance. (**D**) No significant changes in cone ERG amplitude and sensitivity of bipolar cells in ω-3 versus ω-6 LCPUFA fed mice. Representative cone ERG plots are shown. *n* = 13–14 eyes. Multiple *t*-test. n.s., no significance. (**E**) Decreased body growth in ω-3 versus ω-6 LCPFA-fed mice. *n* = 8 mice per group. Unpaired *t*-test, ** *p* < 0.01.

To gain further insight into the long-term impact of ω-6 and ω-3 LCPUFA on retinal development, we evaluated retinal neuronal function with ERG in ω-3 vs. ω-6 LCPUFA-fed C57BL/6J mice at P30 after inducing hyperglycemia with STZ (25mg/kg, daily i.p. from P2 to 12, Figure 7A). We found that the mouse survival rate using higher doses of STZ (50 mg/kg (i.p. from P1 to 9)) was low at P30. The a-wave is a measure of photoreceptor function while the b-wave is a measure of mostly bipolar cell function (Figure 7B) [56]. We found that the ω-3 vs. the ω-6 LCPUFA diet decreased rod pathway responses in Phase I ROP mice (Figure 7C). The cone pathway responses were less impacted (Figure 7D). Furthermore, we observed a lower body weight in ω-3 vs. ω-6 LCPUFA diet at P30 (Figure 7E). These findings suggested that maintaining adequate ω-6 LCPUFA levels is essential in retinal development and overall body growth, in line with our finding that ω-6 LCPUFA promoted neural retinal maturation and metabolism (Figure 4). 

### 3.5. Dietary ω-3 LCPUFA Increased APN and APN Receptor Levels

**Figure 8:**
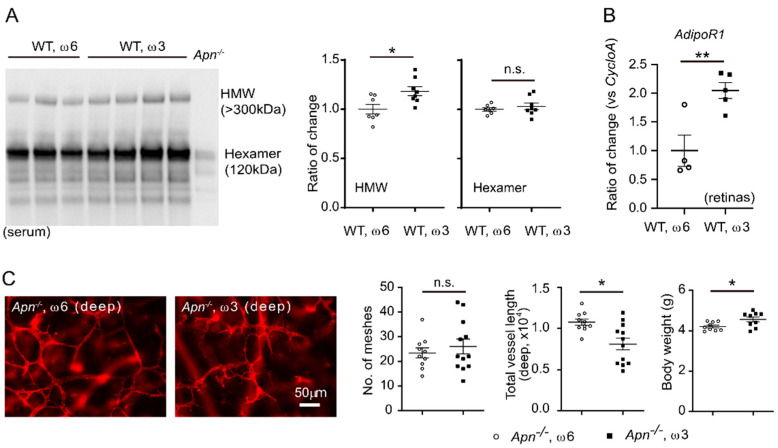
APN mediated ω-3-LCPUFA promotion on retinal vessel growth in Phase I ROP. (**A**) ω- 3 versus ω-6 LCPUFA-enriched diet increased serum HMW APN levels. A total of 1 μL serum was used for Western Blot. *n* = 7–8 mice per group. Unpaired *t*-test. * *p* < 0.05, n.s., no significance. Data are represented as mean ± SEM. (**B**) ω-3 versus ω-6 LCPUFA-enriched diet increased retinal *AdipoR1* expression. *n* = 4–5 mice per group. Unpaired *t*-test. ** *p* < 0.01. Data are represented as mean ± SEM. (**C**) APN deficiency partially abolished ω-3 vs. ω-6 LCPUFA promotion of retinal vessel growth. Representative images of retinal vessels in P10 *Apn^−/−^* Phase I ROP mice fed a ω-3 or ω-6 LCPUFA-enriched diet from P6. Retinal vessels were stained with isolectin. Scale bar, 50 μm. Loss of APN delayed vascular network formation in mice fed with either diet. *n* = 10–12 eyes per group. Loss of APN also delayed body growth in ω-6 LCPUFA-fed mice. *n* = 9 mice per group. Unpaired *t*-test. * *p* < 0.05, n.s., no significance. Data are represented as mean ± SEM.

Dietary ω-3 LCPUFA supplementation increased APN production and secretion from white adipose tissue in mice modeling late phase ROP [28]. In this study, we examined serum APN levels at P10 in the mouse Phase I ROP model. Circulating bioactive high-molecular-weight (HMW) APN was increased in ω-3 vs. ω-6 LCPUFA-fed C57BL/6J mice (Figure 8A). In addition, we also found that ω-3 vs. ω-6 LCPUFA supplementation increased retinal APN receptor *AdipoR1* mRNA levels in Phase I ROP retinas at P10 (Figure 8B). These findings suggested that ω-3 LCPUFA might activate the APN pathway in the Phase I ROP model. Loss of APN partially abolished the protective effects of ω-3 LCPUFA on retinal vascular patterning (Figure 8C). We also observed higher body weight in ω-3 LCPUFA-fed *Apn^−/−^* mice, further suggesting that the delayed retinal vascular patterning was not due to overall body growth. This finding suggested that APN is involved in mediating ω-3 LCPUFA’s promotion of deep retinal vessel growth in the Phase I ROP model.

### 3.6. Loss of APN Decreased Retinal Lipid Composition

**Figure 9:**
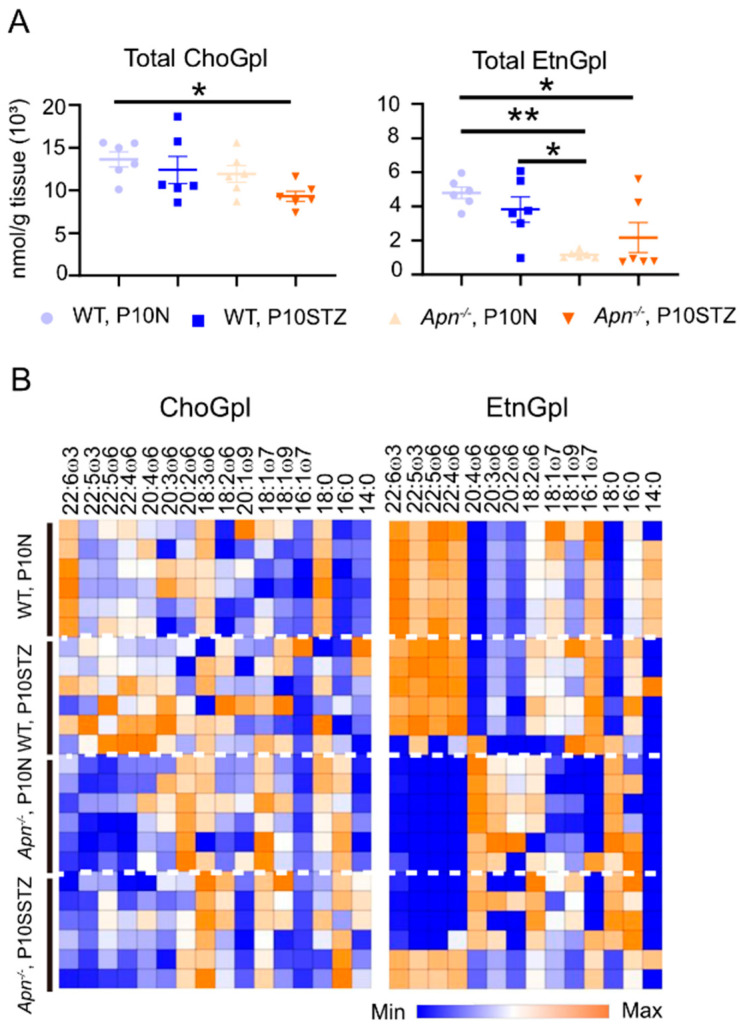
Loss of APN decreased unsaturated retinal lipid composition. Hyperglycemic induction in C57BL/6J (WT) and *Apn^−/−^* mice. An amount of 50 mg/kg STZ was i.p. injected daily from P1 to P9. At P10 the retinas were collected for lipidomic analysis. (**A**) Loss of APN caused reduced total fatty acids in ChoGpl and EtnGpl. ChoGpl, phosphatidylcholine; EtnGpl, phosphatidylethanolamine. 6–8 retinas were pooled as *n* = 1. *n* = 6 per group. ANOVA with Tukey’s multiple comparison test. * *p* < 0.05, ** *p* < 0.01. (**B**) In ChoGpl and EtnGpl, loss of APN decreased VLCPUFA (≥22C). 6–8 retinas were pooled as *n* = 1. *n* = 6 per group. Blue: minimum of levels; orange: maximum of levels. Heatmap was generated using ratio of change in percent mol (Morpheus, Broad Institute).

To further examine if low APN levels affect retinal lipid composition in Phase I ROP, we performed lipidomics analysis. At P10, retinas were collected from normoglycemic and hyperglycemic conditions. The retina lipid composition was examined for the major phospholipid classes ChoGpl and EtnGpl, which make up 80% of total retinal lipids in the rod outer segment [57,58,59,60]. Overall, total fatty acids in ChoGpl were decreased with the loss of APN with hypreglycemia and total fatty acids in EtnGpl were decreased with the loss of APN under both normoglycemia and hyperglycemia (Figure 9A). Specifically, loss of APN decreased very-long-chain polyunsaturated fatty acids (≥22C, VLCPUFA), including ω-3 (docosapentaenoic acid DPA 22:5 ω-3, DHA 22:6 ω-3), and ω-6 (adrenic acid AdA 22:4 ω-6, DPA 22:5 ω-6) (Figure 9B). These results further confirmed that low APN levels are associated with low retinal ω-3/ω-6 LCPUFA. 

## 4. Discussion

We found that dietary ω-3 LCPUFA (DHA and EPA) promoted early retinal vascular development, while ω-6 LCPUFA (AA) was essential in maintaining retinal neuronal development and metabolism. Loss of APN led to less ω-3 LCPUFA protection on retinal vascularization and an unbalanced retinal unsaturated lipid composition in hyperglycemia-associated Phase I ROP (delayed vascular development). 

Some clinical trials showed that fish-oil (rich in ω-3 LCPUFA) supplementation reduces the risk for severe ROP in premature infants [19,20,21]. However, other studies found no benefit of fish-oil supplementation on ROP outcome [22,26]. Furthermore, fish-oil supplementation or dietary intake of food rich in ω-3 LCPUFA decreases the risk for age-related macular degeneration [61,62,63,64]. However, dietary supplementation of DHA (350 mg per day) and EPA (650 mg per day) did not reduce the risk of progressing from moderate to advanced age-related macular degeneration, beyond the effects of the original AREDS formulation within the health conscious and well-nourished AREDS2 cohort [65]. Nonetheless, the companies that supplied the NEI AREDS2 project with the study supplements now produce formulations combining DHA and EPA with the AREDS2 formulation endorsed by the NEI. The reasons for the inconsistent observations of ω-3 LCPUFA supplementation in preventing retinopathies are still unclear. It has been noted that fish-oil supplementation (rich in ω-3 LCPUFA) may lower circulating AA levels [66,67] and decrease the AA to DHA ratio in premature infants [22]. Recent reports showed that low postnatal levels of serum AA are strongly associated with ROP development [68], and maintaining adequate AA levels is required for the ω-3 LCPUFA to protect against ROP [23]. Oral supplementation of DHA and AA at 1:2 ratio improves visual acuity at 12 months of age in premature infants, but a higher DHA/AA ratio does not show any additional improvement [24]. More recently, it was shown that oral DHA and AA at 1:2 ratio reduces severe ROP by 50% and increases the serum levels of both AA and DHA in extremely preterm infants [69]. Higher daily DHA ingestion may be associated with less severe ROP only in infants with sufficiently high AA levels [23]. These observations suggest that maintaining DHA/AA equilibrium is essential in protecting against retinal diseases. 

Low levels of circulating DHA are associated with low APN levels in premature infants [28]. Low APN levels are associated with delayed retinal vascularization in Phase I ROP and increase the risk for vision-threatening Phase II ROP [15,28]. In mice, dietary DHA and EPA promote normal retinal vascularization and inhibit pathologic retinal angiogenesis in neonates modeling proliferative ROP [27,28,33,70]. Furthermore, loss of *AdipoR1* attenuates retinal uptake, conservation and elongation of DHA, and causes photoreceptor degeneration in mice [71]. These observations suggest that unbalanced retinal LCPUFA levels cause retinal neurovascular dysfunction. We observed that a ω-3 LCPUFA (DHA and EPA, no AA) diet better protected against hyperglycemia-associated retinal vascular growth delay than a ω-6 LCPUFA (AA, no DHA and EPA) diet at P10. This finding suggests that increasing ω-3 LCPUFA levels would benefit retinal vascularization in early ROP, to prevent late severe ROP by increasing the vascularized retinal area thereby decreasing stimuli for neovascularization. These protective effects are possibly due to increased circulating APN levels and retinal production of *AdipoR1*, because the APN pathway regulates platelet-derived growth factor B production in photoreceptors to control retinal vessel growth in Phase I ROP mice [15]. In the current study, we additionally found that loss of APN led to an unbalanced retinal lipid composition with a robust decrease of ω-3 and ω-6 LCPUFA levels in ChoGpl and EtnGpl. Our finding suggested that mice with APN deficiency have disrupted retinal lipid composition, contributing to disrupted ω-3/ ω-6 equilibrium and delayed retinal development. 

In the current study, using single-cell transcriptomics, we found that the ω-6 (AA) vs. ω-3 (DHA/EPA) diet had lower *Igf1* and *Igf1r* levels in retinal neuronal cells, which was validated in total retinas with qPCR. The *Igf1* signaling is essential in preserving retinal vascularization in Phase 1 ROP [72,73,74,75,76,77]. We also examined if ω-3 vs. ω-6 LCPUFA modulates the expression of genes involved in cell death; we found very mild changes in *Bcl2, Bcl2l1, Bcl2l11 Casp*, and *Myc* in the retinal clusters (data not shown). Overall, we did not identify any significant pathways using the genes with *p* < 0.05 in the endothelial cell cluster, possibly because the total cell number in this cluster was too small. We do not exclude possible direct effects of DHA on retinal endothelial cell function. It was reported that DHA increases antioxidant capacity of human umbilical vein endothelial cells *in vitro* [78]. 

Interestingly, we observed that an ω-6 (AA) vs. ω-3 (DHA/EPA) diet accelerates the retinal neuronal maturing process along with inducing an increased metabolism to balance the energy demand and supply in retinal neurons. Improving retinal metabolism protects against retinal vascular and neuronal developmental dysfunction in the Phase I ROP mouse model [15]. Therefore, we speculated that an ω-3 LCPUFA diet with concomitant low AA shortage might lead to long-term retinal abnormalities, although better retinal vascular coverage was observed at P10. We found that at P30, Phase I ROP mice fed an ω-3 LCPUFA diet with AA shortage exhibited worse rod photoreceptor and downstream bipolar cell responses than those fed an ω-6 LCPUFA diet with ω-3 LCPUFA deficits. Taken together, our findings suggested that AA is essential in maintaining retinal metabolism and long-term retinal neuronal development. 

## 5. Conclusions

In summary, we show that in mice modeling early Phase I ROP with delayed vascular development and metabolic dysfunction, ω-3 LCPUFA (DHA and EPA) was important in promoting normal retinal vascularization, while ω-6 LCPUFA (AA) was essential in maintaining retinal metabolism and neuronal development. ω-3 LCPUFA’s promotion of retinal vessel growth was partially mediated by APN and loss of APN caused an unbalanced retinal LCPUFA (ω-3 and ω-6) composition, which was associated with retinal vascular and neuronal dysfunction. We did not observe significant differences in blood glucose levels between ω-3 and ω-6 LCPUFA-fed mice, suggesting the impact of LCPUFA might not through modulate circulating hyperglycemia. An increase in retinal *Igf1, Igf1r*, and *Adipor1* gene expression suggested a possible direct impact of ω-3 vs. ω-6 LCPUFA on retinal health. Taken together, our current findings suggested that it is important to maintain adequate ω-6 LCPUFA (AA) levels while supplementing with ω-3 LCPUFA to prevent ROP. 

## Data Availability

The datasets generated during and/or analyzed during the current study are available from the corresponding author on reasonable request.

## Supplementary Materials

The following supporting information can be downloaded at: https://www.mdpi.com/article/10.3390/nu14071333/s1, Table S1: Composition of ω-3 and ω-6 diets. Figure S1: Dietary ω-3 vs. ω-6 LCPUFA supplementation from birth under normoglycemia. Figure S2: The most up-regulated genes in cell cluster of Phase I ROP versus normal control mice; Figure S3: The most down-regulated genes in cell cluster of Phase I ROP versus normal control mice.

## Abbreviations

AA	Arachidonic acid
AdA	Adrenic acid
APN	Adiponectin
ATP	Adenosine triphosphate
cAMP	Cyclic adenosine monophosphate
ChoGpl	Choline glycerophospholipids
DHA	Docosahexaenoic acid
DPA	Docosapentaenoic acid
ECL	Enhanced chemiluminescence
EPA	Eicosapentaenoic acid
ERG	Electroretinography
EtnGpl	Ethanolamine glycerophospholipids
FAME	Fatty acid methyl esters
GEO	Gene Expression Omnibus
GTP	Guanosine triphosphate
HMS	Harvard Medical School
HMW	High-molecular-weight
HVGs	High variable genes
IGF1	Insulin-like growth factor 1
LCPUFA	Long-chain polyunsaturated fatty acids
NADH	Nicotinamide adenine dinucleotide (reduced)
PBS	Phosphate buffered saline
ROP	Retinopathy of prematurity
STZ	Streptozotocin
TLC	Thin-layer chromatography
VLCPUFA	Very long-chain polyunsaturated fatty acid
WT	Wild type

## References

[1] Hellstrom A., Smith L.E., Dammann O. (2013). Retinopathy of prematurity. Lancet.

[2] Chen J., Stahl A., Hellstrom A., Smith L.E. (2011). Current update on retinopathy of prematurity: Screening and treatment. Curr. Opin. Pediatr..

[3] Asano M.K., Dray P.B. (2014). Retinopathy of prematurity. Dis. Mon. DM.

[4] Azad R., Dave V., Jalali S. (2011). Use of intravitreal anti-VEGF: Retinopathy of prematurity surgeons’ in Hamlet’s dilemma?. Indian J. Ophthalmol..

[5] Au S.C., Tang S.M., Rong S.S., Chen L.J., Yam J.C. (2015). Association between hyperglycemia and retinopathy of prematurity: A systemic review and meta-analysis. Sci. Rep..

[6] Ahmadpour-Kacho M., Motlagh A.J., Rasoulinejad S.A., Jahangir T., Bijani A., Pasha Y.Z. (2014). Correlation between hyperglycemia and retinopathy of prematurity. Pediatr. Int..

[7] Garg R., Agthe A.G., Donohue P.K., Lehmann C.U. (2003). Hyperglycemia and retinopathy of prematurity in very low birth weight infants. J. Perinatol..

[8] Mohamed S., Murray J.C., Dagle J.M., Colaizy T. (2013). Hyperglycemia as a risk factor for the development of retinopathy of prematurity. BMC Pediatr..

[9] Kaempf J.W., Kaempf A.J., Wu Y., Stawarz M., Niemeyer J., Grunkemeier G. (2011). Hyperglycemia, insulin and slower growth velocity may increase the risk of retinopathy of prematurity. J. Perinatol..

[10] Mohsen L., Abou-Alam M., El-Dib M., Labib M., Elsada M., Aly H. (2014). A prospective study on hyperglycemia and retinopathy of prematurity. J. Perinatol..

[11] Ertl T., Gyarmati J., Gaal V., Szabo I. (2006). Relationship between hyperglycemia and retinopathy of prematurity in very low birth weight infants. Biol. Neonate.

[12] Chavez-Valdez R., McGowan J., Cannon E., Lehmann C.U. (2011). Contribution of early glycemic status in the development of severe retinopathy of prematurity in a cohort of ELBW infants. J. Perinatol..

[13] Binder N.D., Raschko P.K., Benda G.I., Reynolds J.W. (1989). Insulin infusion with parenteral nutrition in extremely low birth weight infants with hyperglycemia. J. Pediatr..

[14] Blanco C.L., Baillargeon J.G., Morrison R.L., Gong A.K. (2006). Hyperglycemia in extremely low birth weight infants in a predominantly Hispanic population and related morbidities. J. Perinatol..

[15] Fu Z., Lofqvist C.A., Liegl R., Wang Z., Sun Y., Gong Y., Liu C.H., Meng S.S., Burnim S.B., Arellano I. (2018). Photoreceptor glucose metabolism determines normal retinal vascular growth. EMBO Mol. Med..

[16] Salis E.R., Reith D.M., Wheeler B.J., Broadbent R.S., Medlicott N.J. (2017). Hyperglycaemic preterm neonates exhibit insulin resistance and low insulin production. BMJ Paediatr. Open.

[17] Martin C.R., Dasilva D.A., Cluette-Brown J.E., Dimonda C., Hamill A., Bhutta A.Q., Coronel E., Wilschanski M., Stephens A.J., Driscoll D.F. (2011). Decreased postnatal docosahexaenoic and arachidonic acid blood levels in premature infants are associated with neonatal morbidities. J. Pediatr..

[18] Baack M.L., Puumala S.E., Messier S.E., Pritchett D.K., Harris W.S. (2015). What is the relationship between gestational age and docosahexaenoic acid (DHA) and arachidonic acid (ARA) levels?. Prostaglandins Leukot. Essent. Fat Acids.

[19] Pawlik D., Lauterbach R., Walczak M., Hurkala J., Sherman M.P. (2014). Fish-oil fat emulsion supplementation reduces the risk of retinopathy in very low birth weight infants: A prospective, randomized study. JPEN J. Parenter. Enter. Nutr..

[20] Pawlik D., Lauterbach R., Turyk E. (2011). Fish-oil fat emulsion supplementation may reduce the risk of severe retinopathy in VLBW infants. Pediatrics.

[21] Beken S., Dilli D., Fettah N.D., Kabatas E.U., Zenciroglu A., Okumus N. (2014). The influence of fish-oil lipid emulsions on retinopathy of prematurity in very low birth weight infants: A randomized controlled trial. Early Hum. Dev..

[22] Najm S., Lofqvist C., Hellgren G., Engstrom E., Lundgren P., Hard A.L., Lapillonne A., Savman K., Nilsson A.K., Andersson M.X. (2017). Effects of a lipid emulsion containing fish oil on polyunsaturated fatty acid profiles, growth and morbidities in extremely premature infants: A randomized controlled trial. Clin. Nutr. ESPEN.

[23] Hellstrom A., Pivodic A., Granse L., Lundgren P., Sjobom U., Nilsson A.K., Soderling H., Hard A.L., Smith L.E.H., Lofqvist C.A. (2021). Association of Docosahexaenoic Acid and Arachidonic Acid Serum Levels With Retinopathy of Prematurity in Preterm Infants. JAMA Netw Open.

[24] Birch E.E., Carlson S.E., Hoffman D.R., Fitzgerald-Gustafson K.M., Fu V.L., Drover J.R., Castaneda Y.S., Minns L., Wheaton D.K., Mundy D. (2010). The DIAMOND (DHA Intake And Measurement Of Neural Development) Study: A double-masked, randomized controlled clinical trial of the maturation of infant visual acuity as a function of the dietary level of docosahexaenoic acid. Am. J. Clin. Nutr..

[25] Smithers L.G., Gibson R.A., McPhee A., Makrides M. (2008). Higher dose of docosahexaenoic acid in the neonatal period improves visual acuity of preterm infants: Results of a randomized controlled trial. Am. J. Clin. Nutr..

[26] Molloy C.S., Stokes S., Makrides M., Collins C.T., Anderson P.J., Doyle L.W. (2016). Long-term effect of high-dose supplementation with DHA on visual function at school age in children born at <33 wk gestational age: Results from a follow-up of a randomized controlled trial. Am. J. Clin. Nutr..

[27] Fu Z., Liegl R., Wang Z., Gong Y., Liu C.H., Sun Y., Cakir B., Burnim S.B., Meng S.S., Lofqvist C. (2017). Adiponectin Mediates Dietary Omega-3 Long-Chain Polyunsaturated Fatty Acid Protection Against Choroidal Neovascularization in Mice. Investig. Ophthalmol. Vis. Sci..

[28] Fu Z., Lofqvist C.A., Shao Z., Sun Y., Joyal J.S., Hurst C.G., Cui R.Z., Evans L.P., Tian K., SanGiovanni J.P. (2015). Dietary omega-3 polyunsaturated fatty acids decrease retinal neovascularization by adipose-endoplasmic reticulum stress reduction to increase adiponectin. Am. J. Clin. Nutr..

[29] Ito R., Satoh-Asahara N., Yamakage H., Sasaki Y., Odori S., Kono S., Wada H., Suganami T., Ogawa Y., Hasegawa K. (2014). An increase in the EPA/AA ratio is associated with improved arterial stiffness in obese patients with dyslipidemia. J. Atheroscler. Thromb..

[30] Crawford M.A., Golfetto I., Ghebremeskel K., Min Y., Moodley T., Poston L., Phylactos A., Cunnane S., Schmidt W. (2003). The potential role for arachidonic and docosahexaenoic acids in protection against some central nervous system injuries in preterm infants. Lipids.

[31] Martinez M. (1992). Tissue levels of polyunsaturated fatty acids during early human development. J. Pediatr..

[32] Dorrell M.I., Friedlander M. (2006). Mechanisms of endothelial cell guidance and vascular patterning in the developing mouse retina. Prog. Retin. Eye Res..

[33] Connor K.M., SanGiovanni J.P., Lofqvist C., Aderman C.M., Chen J., Higuchi A., Hong S., Pravda E.A., Majchrzak S., Carper D. (2007). Increased dietary intake of omega-3-polyunsaturated fatty acids reduces pathological retinal angiogenesis. Nat. Med..

[34] Gong Y., Fu Z., Edin M.L., Liu C.H., Wang Z., Shao Z., Fredrick T.W., Saba N.J., Morss P.C., Burnim S.B. (2016). Cytochrome P450 Oxidase 2C Inhibition Adds to omega-3 Long-Chain Polyunsaturated Fatty Acids Protection Against Retinal and Choroidal Neovascularization. Arterioscler. Thromb. Vasc. Biol..

[35] Gong Y., Shao Z., Fu Z., Edin M.L., Sun Y., Liegl R.G., Wang Z., Liu C.H., Burnim S.B., Meng S.S. (2016). Fenofibrate Inhibits Cytochrome P450 Epoxygenase 2C Activity to Suppress Pathological Ocular Angiogenesis. EBioMedicine.

[36] Fu Z., Qiu C., Cagnone G., Tomita Y., Huang S., Cakir B., Kotoda Y., Allen W., Bull E., Akula J.D. (2021). Retinal glial remodeling by FGF21 preserves retinal function during photoreceptor degeneration. iScience.

[37] Tomita Y., Qiu C., Bull E., Allen W., Kotoda Y., Talukdar S., Smith L.E.H., Fu Z. (2021). Muller glial responses compensate for degenerating photoreceptors in retinitis pigmentosa. Exp. Mol. Med..

[38] Zilionis R., Nainys J., Veres A., Savova V., Zemmour D., Klein A.M., Mazutis L. (2017). Single-cell barcoding and sequencing using droplet microfluidics. Nat. Protoc..

[39] Klein A.M., Mazutis L., Akartuna I., Tallapragada N., Veres A., Li V., Peshkin L., Weitz D.A., Kirschner M.W. (2015). Droplet barcoding for single-cell transcriptomics applied to embryonic stem cells. Cell.

[40] Srinivasa S., Garcia-Martin R., Torriani M., Fitch K.V., Carlson A.R., Kahn C.R., Grinspoon S.K. (2021). Altered pattern of circulating miRNAs in HIV lipodystrophy perturbs key adipose differentiation and inflammation pathways. JCI Insight.

[41] Hung R.J., Hu Y., Kirchner R., Liu Y., Xu C., Comjean A., Tattikota S.G., Li F., Song W., Ho Sui S. (2020). A cell atlas of the adult Drosophila midgut. Proc. Natl. Acad. Sci. USA.

[42] Svensson V., Natarajan K.N., Ly L.H., Miragaia R.J., Labalette C., Macaulay I.C., Cvejic A., Teichmann S.A. (2017). Power analysis of single-cell RNA-sequencing experiments. Nat. Methods.

[43] Srivastava A., Sarkar H., Gupta N., Patro R. (2016). RapMap: A rapid, sensitive and accurate tool for mapping RNA-seq reads to transcriptomes. Bioinformatics.

[44] Stuart T., Butler A., Hoffman P., Hafemeister C., Papalexi E., Mauck W.M., Hao Y., Stoeckius M., Smibert P., Satija R. (2019). Comprehensive Integration of Single-Cell Data. Cell.

[45] Yan W., Laboulaye M.A., Tran N.M., Whitney I.E., Benhar I., Sanes J.R. (2020). Mouse Retinal Cell Atlas: Molecular Identification of over Sixty Amacrine Cell Types. J. Neurosci. Off. J. Soc. Neurosci..

[46] Finak G., McDavid A., Yajima M., Deng J., Gersuk V., Shalek A.K., Slichter C.K., Miller H.W., McElrath M.J., Prlic M. (2015). MAST: A flexible statistical framework for assessing transcriptional changes and characterizing heterogeneity in single-cell RNA sequencing data. Genome Biol..

[47] Fu Z., Wang Z., Liu C.H., Gong Y., Cakir B., Liegl R., Sun Y., Meng S.S., Burnim S.B., Arellano I. (2018). Fibroblast Growth Factor 21 Protects Photoreceptor Function in Type 1 Diabetic Mice. Diabetes.

[48] Hood D.C., Birch D.G. (1994). Rod phototransduction in retinitis pigmentosa: Estimation and interpretation of parameters derived from the rod a-wave. Investig. Ophthalmol. Vis. Sci..

[49] Lamb T.D., Pugh E.N. (1992). A quantitative account of the activation steps involved in phototransduction in amphibian photoreceptors. J. Physiol..

[50] Pugh E.N., Lamb T.D. (1993). Amplification and kinetics of the activation steps in phototransduction. Biochim. Biophys. Acta.

[51] Fulton A.B., Rushton W.A. (1978). The human rod ERG: Correlation with psychophysical responses in light and dark adaptation. Vis. Res..

[52] Akula J.D., Mocko J.A., Benador I.Y., Hansen R.M., Favazza T.L., Vyhovsky T.C., Fulton A.B. (2008). The neurovascular relation in oxygen-induced retinopathy. Mol. Vis..

[53] Young R.W. (1985). Cell differentiation in the retina of the mouse. Anat. Rec..

[54] Le Y.Z. (2017). VEGF production and signaling in Muller glia are critical to modulating vascular function and neuronal integrity in diabetic retinopathy and hypoxic retinal vascular diseases. Vis. Res..

[55] Zhang J.L., Chen G.W., Liu Y.C., Wang P.Y., Wang X., Wan Y.L., Zhu J., Gao H.Q., Yin J., Wang W. (2012). Secreted protein acidic and rich in cysteine (SPARC) suppresses angiogenesis by down-regulating the expression of VEGF and MMP-7 in gastric cancer. PLoS ONE.

[56] Akula J.D., Hansen R.M., Tzekov R., Favazza T.L., Vyhovsky T.C., Benador I.Y., Mocko J.A., McGee D., Kubota R., Fulton A.B. (2010). Visual cycle modulation in neurovascular retinopathy. Exp. Eye Res..

[57] Fu Z., Chen C.T., Cagnone G., Heckel E., Sun Y., Cakir B., Tomita Y., Huang S., Li Q., Britton W. (2019). Dyslipidemia in retinal metabolic disorders. EMBO Mol. Med..

[58] Fu Z., Kern T.S., Hellstrom A., Smith L. (2020). Fatty acid oxidation and photoreceptor metabolic needs. J. Lipid Res..

[59] Daemen F.J. (1973). Vertebrate rod outer segment membranes. Biochim. Biophys. Acta.

[60] Anderson R.E., Maude M.B. (1970). Phospholipids of bovine outer segments. Biochemistry.

[61] Sangiovanni J.P., Agron E., Meleth A.D., Reed G.F., Sperduto R.D., Clemons T.E., Chew E.Y., Age-Related Eye Disease Study Research Group (2009). {omega}-3 Long-chain polyunsaturated fatty acid intake and 12-y incidence of neovascular age-related macular degeneration and central geographic atrophy: AREDS report 30, a prospective cohort study from the Age-Related Eye Disease Study. Am. J. Clin. Nutr..

[62] Christen W.G., Schaumberg D.A., Glynn R.J., Buring J.E. (2011). Dietary omega-3 fatty acid and fish intake and incident age-related macular degeneration in women. Arch. Ophthalmol..

[63] Tan J.S., Wang J.J., Flood V., Mitchell P. (2009). Dietary fatty acids and the 10-year incidence of age-related macular degeneration: The Blue Mountains Eye Study. Arch. Ophthalmol..

[64] Ho L., van Leeuwen R., Witteman J.C., van Duijn C.M., Uitterlinden A.G., Hofman A., de Jong P.T., Vingerling J.R., Klaver C.C. (2011). Reducing the genetic risk of age-related macular degeneration with dietary antioxidants, zinc, and omega-3 fatty acids: The Rotterdam study. Arch. Ophthalmol.

[65] Age-Related Eye Disease Study 2 Research Group (2013). Lutein + zeaxanthin and omega-3 fatty acids for age-related macular degeneration: The Age-Related Eye Disease Study 2 (AREDS2) randomized clinical trial. JAMA.

[66] Zhao Y., Wu Y., Pei J., Chen Z., Wang Q., Xiang B. (2015). Safety and efficacy of parenteral fish oil-containing lipid emulsions in premature neonates. J. Pediatr. Gastroenterol. Nutr..

[67] D’Ascenzo R., Savini S., Biagetti C., Bellagamba M.P., Marchionni P., Pompilio A., Cogo P.E., Carnielli V.P. (2014). Higher docosahexaenoic acid, lower arachidonic acid and reduced lipid tolerance with high doses of a lipid emulsion containing 15% fish oil: A randomized clinical trial. Clin. Nutr..

[68] Lofqvist C.A., Najm S., Hellgren G., Engstrom E., Savman K., Nilsson A.K., Andersson M.X., Hard A.L., Smith L.E.H., Hellstrom A. (2018). Association of Retinopathy of Prematurity With Low Levels of Arachidonic Acid: A Secondary Analysis of a Randomized Clinical Trial. JAMA Ophthalmol..

[69] Hellstrom A., Nilsson A.K., Wackernagel D., Pivodic A., Vanpee M., Sjobom U., Hellgren G., Hallberg B., Domellof M., Klevebro S. (2021). Effect of Enteral Lipid Supplement on Severe Retinopathy of Prematurity: A Randomized Clinical Trial. JAMA Pediatr..

[70] Stahl A., Sapieha P., Connor K.M., Sangiovanni J.P., Chen J., Aderman C.M., Willett K.L., Krah N.M., Dennison R.J., Seaward M.R. (2010). Short communication: PPAR gamma mediates a direct antiangiogenic effect of omega 3-PUFAs in proliferative retinopathy. Circ. Res..

[71] Rice D.S., Calandria J.M., Gordon W.C., Jun B., Zhou Y., Gelfman C.M., Li S., Jin M., Knott E.J., Chang B. (2015). Adiponectin receptor 1 conserves docosahexaenoic acid and promotes photoreceptor cell survival. Nat. Commun..

[72] Cakir B., Hellstrom W., Tomita Y., Fu Z., Liegl R., Winberg A., Hansen-Pupp I., Ley D., Hellstrom A., Lofqvist C. (2020). IGF1, serum glucose, and retinopathy of prematurity in extremely preterm infants. JCI Insight.

[73] Hellstrom A., Perruzzi C., Ju M., Engstrom E., Hard A.L., Liu J.L., Albertsson-Wikland K., Carlsson B., Niklasson A., Sjodell L. (2001). Low IGF-I suppresses VEGF-survival signaling in retinal endothelial cells: Direct correlation with clinical retinopathy of prematurity. Proc. Natl. Acad. Sci. USA.

[74] Hard A.L., Smith L.E., Hellstrom A. (2013). Nutrition, insulin-like growth factor-1 and retinopathy of prematurity. Semin. Fetal Neonatal Med..

[75] Hellstrom A., Engstrom E., Hard A.L., Albertsson-Wikland K., Carlsson B., Niklasson A., Lofqvist C., Svensson E., Holm S., Ewald U. (2003). Postnatal serum insulin-like growth factor I deficiency is associated with retinopathy of prematurity and other complications of premature birth. Pediatrics.

[76] Jensen A.K., Ying G.S., Huang J., Quinn G.E., Binenbaum G. (2017). Postnatal Serum Insulin-Like Growth Factor I and Retinopathy of Prematurity. Retina.

[77] Hellgren G., Lundgren P., Pivodic A., Lofqvist C., Nilsson A.K., Ley D., Savman K., Smith L.E., Hellstrom A. (2021). Decreased Platelet Counts and Serum Levels of VEGF-A, PDGF-BB, and BDNF in Extremely Preterm Infants Developing Severe ROP. Neonatology.

[78] Ishikado A., Morino K., Nishio Y., Nakagawa F., Mukose A., Sono Y., Yoshioka N., Kondo K., Sekine O., Yoshizaki T. (2013). 4-Hydroxy hexenal derived from docosahexaenoic acid protects endothelial cells via Nrf2 activation. PLoS ONE.

