# Supplementary material for: Omega-3/Omega-6 Long-Chain Fatty Acid Imbalance in Phase I Retinopathy of Prematurity
Source: Nutrients. 2022 Mar 23;14(7):1333. doi: 10.3390/nu14071333 (PMC9002570; doi:10.3390/nu14071333)
Supplement: Supplementary file 1 [file nutrients-14-01333-s001.zip › nutrients-1597572-supplementary.pdf]

Supplementary Materials:

Table S1. Composition of ω-3 and ω-6 diets.

Product #	D14040901		D14040902s	
	Omega-3 Diet		Omega-6 Diet	
	gm%	*kcal%*	gm%	*kcal%*
Protein	20	20	20	20
Carbohydrate	61	59	61	59
Fat	10	22	10	22
Total		*100*		*100*
kcal/gm	4.1		4.1	
**Ingredient**	**gm**	***kcal***	**gm**	***kcal***
Casein	200	*800*	200	*800*
L-Cystine	3	*12*	3	*12*
Corn Starch	367	*1468*	367	*1468*
Maltodextrin 10	132	*528*	132	*528*
Sucrose	100	*400*	100	*400*
Cellulose, BW200	50	*0*	50	*0*
Soybean Oil	0	*0*	0	*0*
ARASCO (42% ARA)	0	*0*	4.8	*43*
DHASCO (43% DHA)	1.042	*9*	0	*0*
MEG-3 (45% EPA, 24% DHA)	2.3	*21*	0	*0*
Safflower Oil, High Oleic	96.658	*870*	95.2	*857*
t-Butylhydroquinone	0.014	*0*	0.014	*0*
Ascorbyl Palmitate	0.5	*0*	0.5	*0*
Mineral Mix S10022G	35	*0*	35	*0*
Vitamin Mix V10037	10	*40*	10	*40*
Choline Bitartrate	2.5	*0*	2.5	*0*
**Total**	**1000**	***4148***	**1000**	***4148***

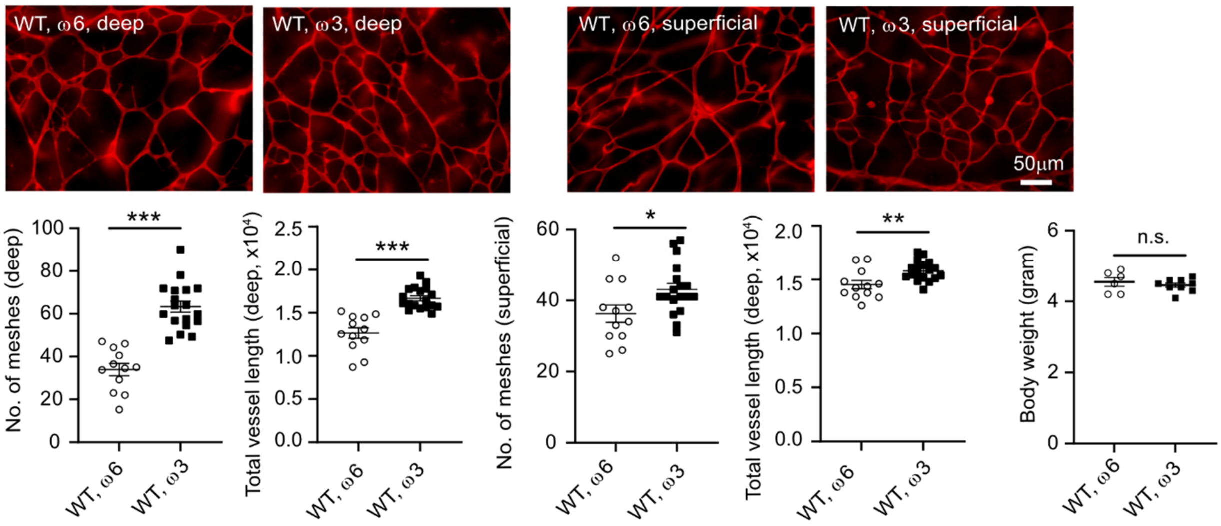

Figure S1. Dietary ω3- vs. ω6-LCPUFA supplementation from birth increased retinal vascular density under normoglycemia. Representative images of retinal vessels in P10 C57BL/6J STZ mice fed on ω3- or ω6-LCPUFA-enriched diet from birth. Retinal vessels were stained with isolectin. Scale bar, 50μm. ω-3- versus ω-6-LCPUFA-enriched diet increased retinal vascular density at P10. n=12-18 eyes. Unpaired t test. *** P<0.001, ** P<0.01, * P<0.05, n.s., no significance. Data was represented as Mean±SEM.

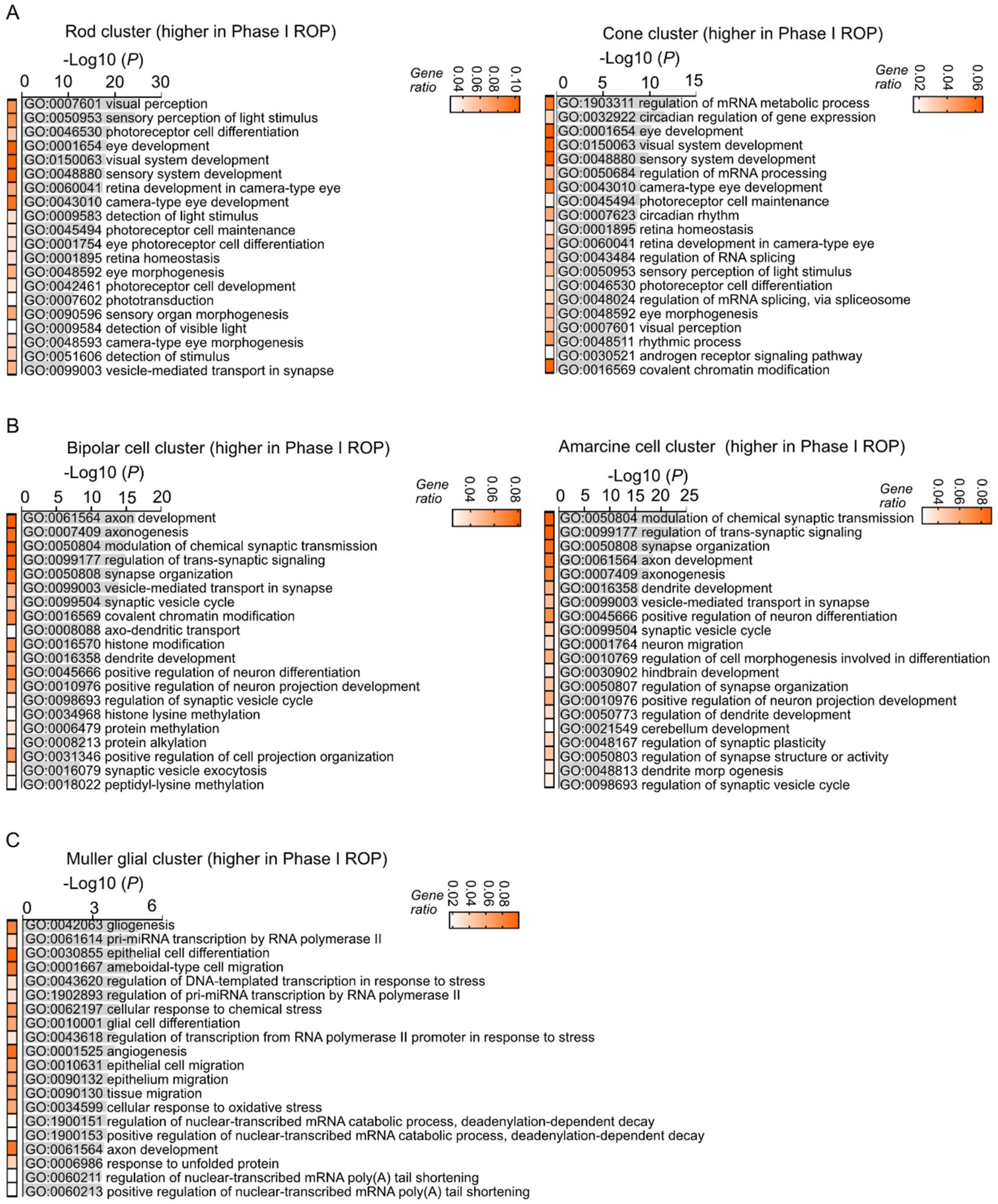

Figure S2. The higher expression of genes in the (**A**) rod and cone, (**B**) bipolar and amacrine cell cluster of Phase I ROP versus normal cotrol mice were associated with visual perception, eye development, axon development, synapse organization related gene-ontology terms. The higher expression of genes in the Müller glial cell cluster (**C**) of Phase I ROP vs normal control mice were associated with gliogenesis, angiogenesis and axon development related gene-ontology terms. Adjusted *P*-values for enriched gene-ontology (GO) terms are showed in bar graphs ($P<0.05$). Gene ratio for each pathway is shown in heatmap.

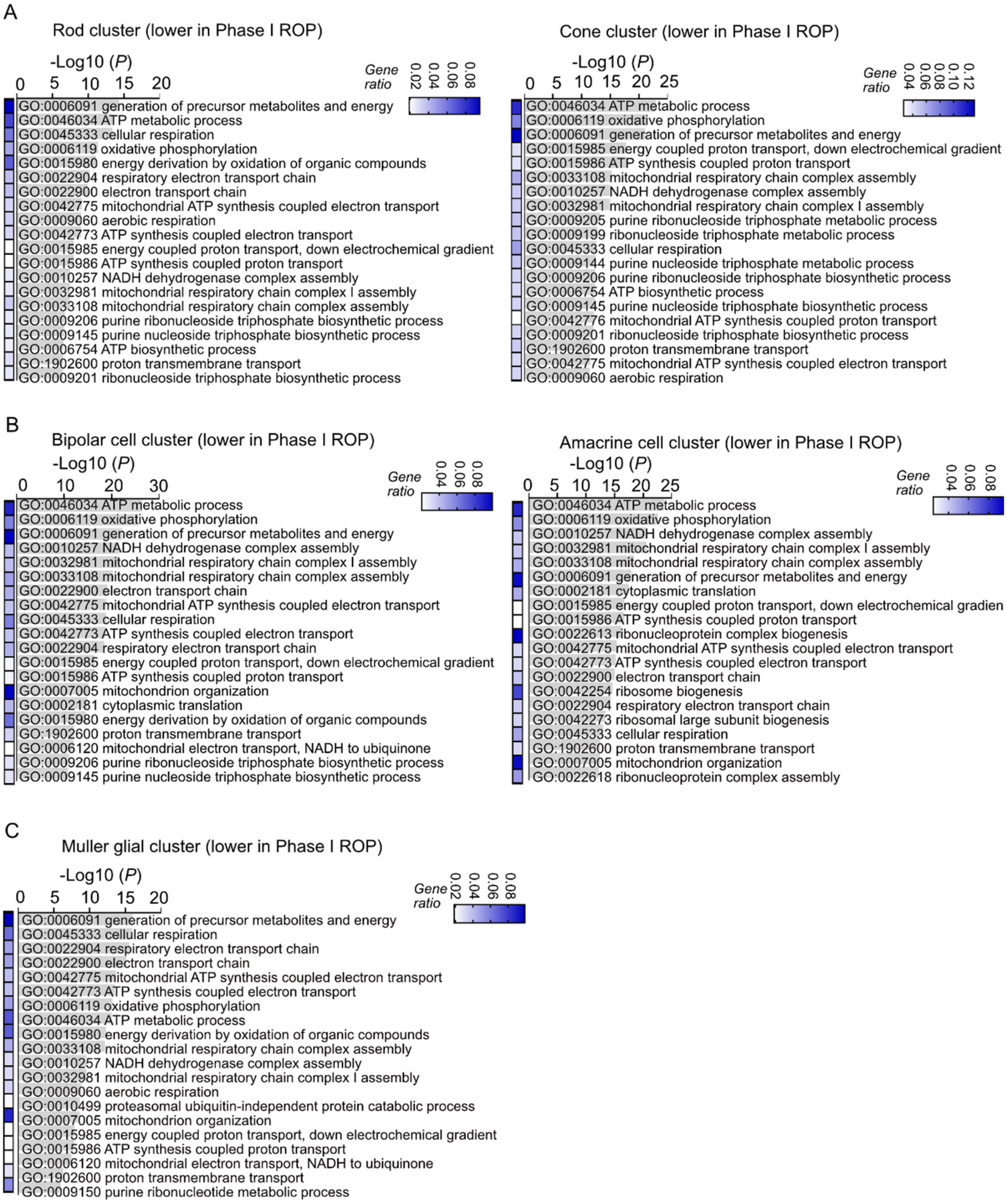

Figure S3. The lower expression of genes (top 20) in the (**A**) rod and cone cluster, (**B)** bipolar and amacrine cell, (**C**) Müller glial cell cluster of Phase I ROP versus normal control retinas were associated with energy production related gene-ontology terms. Adjusted *P*-values for enriched gene-ontology (GO) terms are showed in bar graphs (P<0.05). Gene ratio for each pathway is shown in heatmap.
